# Biodiversity of *Mycobacterium tuberculosis* in Bulgaria Related to Human Migrations or Ecological Adaptation

**DOI:** 10.3390/microorganisms10010146

**Published:** 2022-01-11

**Authors:** Stefan Panaiotov, Dzheni Madzharov, Yordan Hodzhev

**Affiliations:** 1 National Center of Infectious and Parasitic Diseases, 1504 Sofia, Bulgaria; y.hodzhev@ncipd.org; 2 Faculty of History, Sofia University, 1504 Sofia, Bulgaria; madzharov.dzheni@gmail.com

**Keywords:** tuberculosis, biogeography, genotype, history, immigration, adaptation

## Abstract

Bulgaria is among the 18 high-priority countries of the WHO European Region with high rates of tuberculosis. The causative agent of tuberculosis is thought to have emerged in Africa 70,000 years ago, or during the Neolithic age, and colonized the world through human migrations. The established main lineages of tuberculosis correlate highly with geography. The goal of our study was to investigate the biodiversity of *Mycobacterium* *tuberculosis* in Bulgaria in association with human migration history during the last 10 centuries. We analyzed spoligotypes and MIRU-VNTR genotyping data of 655 drug-sensitive and 385 multidrug-resistant *M. tuberculosis* strains collected in Bulgaria from 2008 to 2018. We assigned the genotype of all isolates using SITVITWEB and MIRU-VNTR*plus* databases and software. We investigated the major well-documented historical events of immigration to Bulgaria that occurred during the last millennium. Genetic profiles demonstrated that, with the exceptions of 3 strains of *Mycobacterium bovis* and 18 strains of Lineage 2 (W/Beijing spoligotype), only Lineage 4 (Euro-American) was widely diffused in Bulgaria. Analysis of well-documented immigrations of Roma from the Indian subcontinent during the 10th to the 12th centuries, Turkic peoples from Central Asia in the medieval centuries, and more recently Armenians, Russians, and Africans in the 20th century influenced the biodiversity of *M. tuberculosis* in Bulgaria but only with genotypes of sublineages within the L4. We hypothesize that these sublineages were more virulent, or that ecological adaptation of imported *M. tuberculosis* genotypes was the main driver contributing to the current genetic biodiversity of *M. tuberculosis* in Bulgaria. We also hypothesize that some yet unknown local environmental factors may have been decisive in the success of imported genotypes. The ecological factors leading to local genetic biodiversity in *M. tuberculosis* are multifactorial and have not yet been fully clarified. The coevolution of long-lasting pathogen hosts should be studied, taking into account environmental and ecological changes.

## 1. Introduction

Bulgaria is among the 18 high-priority countries of the WHO European Region with high rates of tuberculosis. TB surveillance in Bulgaria is organized at a national level. In 2008, tuberculosis notification rates in Bulgaria demonstrated 41.2 cases per 100,000 population, with a gradual decline in 2018 to 19.3 cases per 100,000 population (Figure S1). The male/female ratio was 2.0. The hospitalization rate of TB patients was 85–90%. The trend in the treatment success rate for new and relapse TB cases that were treated in 2014–2017 was 83–84%; for patients with multidrug-resistant tuberculosis (MDR-TB), the rate was 50–65%. In 2008 and 2018, the percentages of tuberculosis patients in Bulgaria with pulmonary tuberculosis were 76.4% and 78%, respectively; 43.2% and 48% of the total number of TB cases, were bacteriologically confirmed. The mean age of new native TB cases was 45 years.

More recent TB cases in foreign individuals were not included in this study, but according to statistics, foreign individuals represented 0.2% of the cases in Bulgaria in 2008 and 0.6% of the cases in Bulgaria in 2018.

In 2008, drug susceptibility tests (DST), including tests on susceptibility to isoniazid (INH) and rifampicin (RIF), were performed on 69% of the patients whose tuberculosis was bacteriologically confirmed. In 2018, 89.2% of the tuberculosis patients had a DST test. In 2018, 5% of the tuberculosis cases were patients with pulmonary MDR-TB; of these cases, patients in the age group of 45–54 years were most affected (24.3%). The first XDR-TB case in Bulgaria was confirmed in 2010 [1,2]. 

The causative agent of tuberculosis, *Mycobacterium tuberculosis* (*M. tuberculosis*), probably appeared about 70,000 years ago [3] or during the Neolithic age [4] and spread globally with human migration. The oldest reported case of osteoarticular tuberculosis in Europe was from the region of present-day Hungary [5]. There are no data on when tuberculosis began to spread in Bulgaria. 

*M. tuberculosis* infection leads to various manifestations, including latent infection and/or progression to pulmonary or extrapulmonary tuberculosis. Tuberculosis is a multifactorial disease. Host predisposition, microbial genotype [6], and population migration [7] play major roles in its spread. The species *M. tuberculosis* has some specific features, including the following: (1) *M. tuberculosis* evolved clonally, and horizontal transfer of genetic material has not been proven, or is extremely rare; (2) the evolutionary scenario of *M. tuberculosis* genotypes is one-way and follows a strict hierarchy from old to new branches through successive deletions; and (3) some genotypes are phylogeographically limited, while others are globally distributed.

In recent years, accumulated evidence has indicated that some genetic lineages, sublineages, and genotypes of the *M. tuberculosis* complex (MTBC) evolve in close contact with specific human populations [8,9]. The most important factors in this process are the demography of a population and the host immune response [10]. These selective factors have shaped the phylogeographic structure of the MTBC genotypes we observe around the world today. Nine known lineages (L) of the MTBC, as adapted to humans, are highly correlated with geography. Africa is the only continent harboring all lineages. L1 is predominant around the Indian Ocean (Indo-Oceanic), i.e., among East African-Indian (EAI) spoligotype families. L2 is globally spread but predominant in East Asia, China, Japan, and Russia, i.e., the W/Beijing spoligotype. The East African-Indian L3 lineage, i.e., the Central Asian strain (CAS) spoligotype families, is common in Central Asia. L4, the Euro-American lineage, is globally spread, i.e., in the Latin American-Mediterranean (LAM), Haarlem (H), Anglo-Saxon (X), and some ill-defined T spoligotype families [11]. 

This biodiversity structure is not static. It is related to the dynamics of the migration processes of populations. We also have examples of geographically restricted lineages, e.g., L5 and L6 (known as *Mycobacterium africanum* I and II, respectively) which are distributed only in West Africa [12]. Three MTBC lineages, L7, L8, and L9, are geographically and ecologically restricted within Africa, and are found in Ethiopia, in the Great Lakes region (Rwanda-Uganda) and in East Africa [13,14,15,16].

It has been suggested that major *M. tuberculosis* lineages associate primarily with patients’ geographical origins [8]. Several research teams in large cities, such as San Francisco, London, and Montreal, where immigration and circulation of different genotypes of *M. tuberculosis* are significant, have tested this thesis [17,18]. Studies have shown that circulating genotypes of *M. tuberculosis* among immigrants are predominant in their countries of origin [17]. Among HIV-positive patients with tuberculosis, this relationship is not observed; *M. tuberculosis* in HIV-positive patients can be of any genotype [19]. Studies demonstrate that if a host population is heterogeneous, then each population component could be predisposed to certain genotypes of *M. tuberculosis*, which may be locally associated (the sympatric effect), and the genotypes will not spread among other population groups (the allopatric effect) [20,21]. 

The host, microbial, and ecological factors that contribute to variation in *M. tuberculosis* genotypes are not fully understood. It is assumed that the causes of geographical distribution and biodiversity of *M. tuberculosis* are historical (e.g., via trade, migration, conquest, and globalization) and biological (e.g., via pathogen-host interactions within different, sometimes intermingled, and complex genetic backgrounds), but discussion of environmental factors as drivers for local success or failure in transmission has been limited. 

It is likely that local ecological factors are of primary importance in the transmission of MTBC genotypes to different populations. Data on how environmental factors affect the selection of *M. tuberculosis* genotypes are very limited. Although local adaptation seems to be a plausible model for describing long-term host-pathogen interactions, genotypic evidence for this model was not introduced until recently [22] We can assume, based on competition, that the fittest genotypes would displace others. This assumption is supported currently by the strong likelihood of reduced biodiversity among the L5 and L6 strains in some parts of Africa, due to the presence of the L4 MTBC [23,24].

It is generally believed that by studying the phylogeographic distribution of *M. tuberculosis* genotypes, the historical migration of ethnic groups can be traced [25]. These studies are the subject of paleomicrobiology, which has significantly enriched our knowledge of the history and spread of infectious diseases around the world [26]. Paleomicrobiology has combined microbiology, evolution, history, and anthropology into a common interdisciplinary subject.

How have past mass immigration waves of diverse population groups affected the biodiversity of M. tuberculosis in Bulgaria?

Based on comparative linguistics and historical, anthropogical, and genetic data, it has been established that the world’s Roma population originated in northwestern India, in the regions of Punjab, Rajasthan, and North Guajarat. “Gypsy” is a general term describing several related ethnic groups (even if the word “ethnic” may have different meanings according to different cultural values, including the Roma people). According to the Bulgarian ethnologists E. Marushiakova, V. Popov, A. Pamporov, and I. Tomova, the word “Gypsy” is academically more correct than “Roma,” as it includes both the Roma and similar communities [27,28]. Gypsy communities in different countries have different names. In Bulgaria, they are divided into Kardarashi (Calderashi), Yerlii (old settlers), Rudari (miners), Demirdzhii (blacksmiths), Kopanari (wooden bowl makers), Burgudzhii (drill makers), and others. They are very diverse and cannot be identified as a single population [27,28].

In Europe, Gypsies constitute 3% to 9% of the Romanian, Hungarian, and Bulgarian populations, and they are present in Spain, France, the Czech Republic, Russia, Slovakia, and Serbia [29,30,31,32]. Several Gypsy communities speak different dialects with common origins. Gypsy dialects resemble the Marwari language spoken in Rajasthan, but the dialects diverged from that language. 

Gypsies (i.e., Gypsies, Roma, Tzigane, Gitanos, and Asingani) are considered as a group of nomadic tribes of Indo-Aryan origin. As early as the 5th and 6th centuries, Indian tribes left northern India and headed west, continuing to roam the territories of Pakistan, Afghanistan, Iran, Iraq, and Turkey for several more centuries. Finally, they settled in the Caucasus, the Balkans, Egypt, North Africa, Spain, and Central Europe. They settled in the Balkans in around the 11th century. These historical facts are derived from human genetic studies. A genetic study of 152 members of 13 separate Gypsy communities from across Europe showed that modern Gypsies have common ancestors and originated in northwestern India, which their ancestors left about 1,500 years ago, entering Europe from approximately 700 A.D. to 900 A.D. via the Balkans, the southern Russian steppes, the coast of North Africa, and the Iberian Peninsula, [30]. Other studies have confirmed these observations [29,31,32].

According to the National Statistical Institute, the number of Gypsies in Bulgaria in 2011 was 325,000 [33]. According to Council of Europe statistics, the current number of Gypsies in Bulgaria is probably between 700,000 and 800,000 [34]. The different estimates are probably due to some Gypsies declaring themselves to be Bulgarians or Turks.

The total number of Gypsies in Europe is estimated at around 10 million to 12 million. They are the largest minority group in Europe. The countries with the largest Gypsy populations are Turkey (2,750,000 or 3.71% of the population), Romania (1,850,000 or 8.56% of the population), Bulgaria (750,000 or 9.74% of the population), Spain (725,000 or 1.6% of the population), and Russia (720,000 or 0.51% of the population). They constitute a significant portion of the populations of Hungary (6.1%), Serbia (2.05%), Slovakia (>2%), and the United States (approximately 1,000,000). There are approximately 500,000 to 800,000 Roma in Brazil, descendants of people who were deported from Portugal in the 17th and 18th centuries.

Approximately half a million Turks live in Bulgaria. In this study, it is important to briefly review the migration of Turkic tribes and the composition of the Turkish people, which developed as a result of a complex history. In the beginning, there was a migration of Seljuk tribes from the northern shores of the Aral Sea. In the 11th century, they gradually conquered the present-day territories of Azerbaijan, western Iran, Mesopotamia, much of Asia Minor, and the Levant, where they established two Turkic states, the Great Seljuk and the Iconian Sultanate. These tribes did not have a single origin, but formed a military-tribal alliance of related Oguz tribes that were part of the Turkic peoples. 

In the conquered lands, they mixed with Byzantines, Thracians, Persians, Jews, Arabs, and others [35,36,37]. The Mongol invasion in the west in the early 13th century drove part of the Turkic peoples from Central Asia. They moved to the lands of western Asia Minor, next to Byzantium. At this time, the Iconian Sultanate disintegrated, allowing the settlers to form many new states, the so-called *Asia Minor beyliks* [38]. In 1299, Ottoman (Osman) I, a tribal leader of a small beylik, established the Ottoman state in northwestern Anatolia, which became a powerful empire a century later. The Ottoman beylik subjugated all other beyliks of Asia Minor and began the gradual conquest of the Byzantine Empire. The Ottoman army conquered all the countries of the Balkan Peninsula and continued its conquests in Central Europe from the 15th century to the 17th century. The young empire gradually expanded its holdings, reaching its peak on three continents and six seas during its heyday in the 16th and 17th centuries, with a total population of about 15 million [35,36,37,38,39]. 

The practice of resettling hundreds of thousands of captives within the empire led to the formation of incredible ethnic diversity. In addition to the conquered Christian population, Greeks, Bulgarians, Serbs, Bosnians, Albanians, and Armenians from the Ottoman Empire were displaced by Vlachs, Moldavians, Hungarians, Austrians, Saxons, Russians, Ukrainians, Cossacks, Poles, and others from Europe. Millions of people were part of the Empire, from Persia to India to the countries of North Africa. The Ottoman Empire became an unprecedented mix of different ethnic and religious communities, numbering more than 25. Modern genetic research shows that a significant part of the 83.6 million population of the Republic of Turkey (in 2020) are not of Turkic origin and share a high degree of variation and admixture [40]. This means that the dynamic migration processes within the Ottoman Empire have led to significant biodiversity of different eastern (L1, L2, and L3) and western (L4) sublineages of *M. tuberculosis*.

The environmental factors that might influence the success or failure of some lineages among the Bulgarian TB population remain unclear. The physical environment and/or still unidentified human genetic factors, in combination with specific genetic factors of *M. tuberculosis* lineages or strains, could predict the success or failure within a human population.

No evidence in the literature indicates whether all MTBC genotypes transmitted by mass migrating populations are equally adapted to new geographical and ecological conditions linked to their hosts. This paper aims to describe the biodiversity of genotypes of *M. tuberculosis* in Bulgaria, keeping in mind the role of human migrations over the past thousand years.

## 2. Materials and Methods

### 2.1. Ethics and Collection of M. tuberculosis Strains

From 2008 to 2018, there were 18,112 cases of pulmonary tuberculosis in Bulgaria. Of these cases, about 44% to 56% (*n* = 10,561) were bacteriologically confirmed. A total of 1041 *M. tuberculosis* isolates collected from across the country were included for genotyping. TB patients of recent foreign origin (<15 years) were not included. We randomly selected 655 strains among the total number of isolates that were drug-susceptible (6%). 

**Figure 1:**
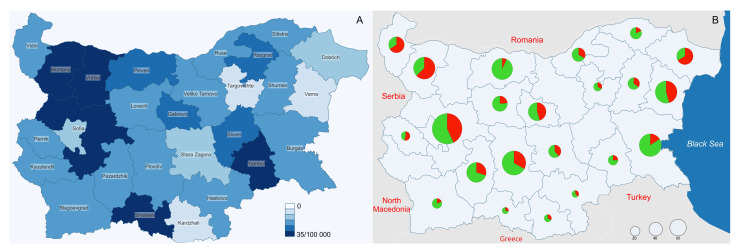
Map of Bulgaria with 28 administrative districts: (**A**) prevalence of new and relapse TB cases (2018); (**B**) sampling sites and proportion of drug sensitive (in green) and multidrug-resistant (in red) MTBC strains. Circle size corresponds to sample size.

Of the multidrug-resistant (MDR) isolates, 386 (80%) were submitted for genotyping (Figure 1). MTBC strains in patients with pulmonary tuberculosis made up 95% of the isolates that were submitted. One isolate per patient was tested for genotyping. Accordingly, our samples characterized the lineage prevalence of tuberculosis in Bulgaria as a whole. Spoligotyping and 24-loci mycobacterial interspersed repetitive units variable number tandem repeat (MIRU-VNTR) assays were used for genotyping *M. tuberculosis*.

The study was conducted in accordance with the Declaration of Helsinki, and approved by the Institutional Review Board/Institutional Ethics Committee (IRB/IEC 00006384) of the National Center of Infectious and Parasitic Diseases, Sofia, Bulgaria (protocol code 1/2018 and date of approval 12 January 2018).

Moreover, patient data are not traceable by any third party other than the National Reference TB Laboratory.

### 2.2. Cultures and DNA Extraction

Isolation of *M. tuberculosis* and phenotypic testing for drug susceptibility to isoniazid 0.1 μg/mL and rifampicin 1 μg/mL were performed at the National Reference TB Laboratory at the National Center of Infectious and Parasitic Diseases, Sofia, Bulgaria by applying the reference BACTEC MGIT960 automated system following the recommendations provided by the manufacturer (Becton Dickinson, Franklin Lakes, NJ, USA).

For DNA isolation, we used a fresh culture grown on a Löwenstein-Jensen medium for 35 days at 37 °C. The DNA was isolated according to the procedure described by van Soolingen [41], including incubations with lysozyme, 10% SDS, proteinase K, 10% cetyltrimethylammonium bromide (CTAB), protein/lipid extraction with phenol/chloroform (1:1 vol), and DNA precipitation with 0.7 vol of isopropanol.

### 2.3. Spoligotyping

Spoligotyping was carried out using a spoligotyping kit (TB-SPOL, Beamedex, Orsay, France) and the methodology developed by Kamerbeek et al. (1997) [42], with the modifications set out below. PCR (25 µL total) was performed for 20–22 cycles with a biotinylated primer DRa (GGTTTTGGGTCTGACGAC) and DRb (CCGAGAGGGGACGGAAAC). The negative control contained dH_2_0, and the positive control contained the DNA of *M. bovis* BCG Sofia 222 and *M.* *tuberculosis* H37Rv. In compliance with the hybridization protocol provided by the supplier, 5 μL of biotinylated PCR product was applied to a freshly prepared microbead working mix in a 96-well flat-bottom plate (Corning Incorporated, New York, NY, USA). Fluorescence produced from streptavidin-phycoerythrin binding in samples and control wells was measured on a flow cytometry-based Luminex 200 system (Bio-Plex, Bio-Rad, Hercules, CA, USA). Median Fluorescence Intensity (MFI) values were obtained via Bio-Plex Manager Software 6.1 version. Raw csv files were converted with Excel spreadsheet files into .xls format to determine positive spacers in each sample and control.

### 2.4. MIRU-VNTR Typing

The methodology, primers, and conditions were as described in the technical manual for MIRU-VNTR genotyping of *M. tuberculosis* developed by Supply [43,44]. Simplex PCR reactions of 24 VNTR regions were performed using primers specific for each flanking VNTR region. PCR products were run on 2% agarose gels. For size determination of the VNTRs, we used 50 bp- and 100 bp-sized markers (Thermo Fisher Scientific, Waltham, MA, USA). The MIRU-VNTR*plus* database and integrated bioinformatics support functions were used as a tool for high-resolution genotyping and clonal identification of *M. tuberculosis* [45].

To define MTBC lineages and sublineages, we applied the spoligotyping-based signatures defined in the SITVIT2 database [12]. Both typing methods, spoligotyping and 24-loci MIRU-VNTR, have proven discriminatory capacities to identify lineages in MTBC. More recent classification schemes based on SNPs, i.e., classification of Coll et al. [46] or Freschi et al. [47], were not possible, as we did not perform a whole-genome sequencing (WGS) analysis in this study and because the criteria for correlation between CRISPR-based and WGS-based classifications of MTBC are still in progress [48].

### 2.5. Historical Documents

We studied documented immigration events to Bulgaria during the last 10 centuries [27,28,29,30,31,32,33,34,35,36,37,38,39,49,50,51,52,53,54,55,56,57,58].

### 2.6. Statistics

SPSS 26 statistical software (IBM, Chicago, IL, USA) was applied for data analysis. A two-way [Sublineage by Drug Susseptibility (DS)] Pearson’s Chi-square test of independence was performed. Counts of DS and MDR for each of the sublineages were compared using probability proportion (expressed in percentages) and its 95% confidence interval (CI), odds ratio (OR), and Pearsons Chi-square (χ2) test with asymptotic significance [59].

## 3. Results

Classical genotyping methods, such as CRISPR-based spoligotyping and 24-loci MIRU-VNTRs, are inexpensive and easy to implement, with standardized analysis. In this sense, they remain helpful in confidently identify TB lineages and genotypes, and are still widely applied.

In total, two lineages (L2 and L4), 23 sublineages, and 108 spoligotypes were identified among the drug-sensitive and multidrug-resistant strains (Table 1). All strains were genotyped by spoligotyping. Thirty-three or 8.5% of the MDR strains were without an MIRU-VNTR result for different reasons, including but not limited to failed typing.

Statistical analysis based on the two-way Pearson’s Chi-square test of independence (Sublineage by Drug Susceptibility) demonstrated an overall association of drug resistance with sublineage (χ_2_(22, 1041) = 46,336; *p* < 0.001). Among the drug-sensitive strains circulating in Bulgaria, the L4/T1 sublineage was 57% and the L4.4/S was 22.15%. Both sublineages accounted for 79% of the drug-sensitive strains. Widely diffused across the country was T1 SIT53 (*n* = 80, 14.5%). Other frequent spoligotypes of the T1 sublineage were SIT154 (5.5%) and SIT284 (4.3%). S-types of SIT34 and SIT125 (both likely belonging to L4.4) accounted for 7.2% (*n* = 47) and 13.5% (*n* = 89) of the drug-sensitive strains. All spoligotypes in the country were randomly diffused.

Among the MDR strains, the sublineage L4.2.2.1/TUR was prevalent, at 51% (199/386). In comparison, among the drug-sensitive strains, the TUR sublineage accounted for less than 2%. MDR occurance probability within the L4.2.2.1/TUR sublineage was 98%, with an odds ratio of 39.80 (Table 2). Hence, the L4.2.2.1/TUR genotype is a marker for MDR tuberculosis in Bulgaria. 

The majority of the MDR TB cases of TUR sublineage are mainly confined to the northwest and northeast districts of the country. MIRU-VNTR analysis demonstrated the distribution of the TUR sublineage strains in 8 clusters, from 3 to >80 strains (data not shown). The L4.2.2.1/TUR sublineage is geographically restricted to the Balkan Peninsula (Albania, Bulgaria, Serbia, and Turkey) and Syria, both of which were part of the Ottoman Empire from the 14th century to the 19th century [1,60,61]. The prevalence in other countries where ethnic groups of Turkic origin live, i.e., Iran (Azeri) or China (Uygurs), is less than 1% [62,63,64].

In Bulgaria, during 2008–2018 the prevalence of the L2/Beijing genotype was low (18/1041; 1.7%). In the Beijing sublineage, 70% were MDR with a probability odds ratio of 2.6. Our previous study demonstrated that the first imported TB case in Bulgaria was from Moldova in 2007–2009 [65]. Not all detected cases of TB caused by the Beijing genotype resulted from human migration. MDR/XDR TB transmission within the country was also observed. Among the prevalent spoligotypes identified in Bulgaria, the L2/Beijing and L4.2.2.1/TUR sublineages demonstrated a higher probability of becoming MDR (Table 2).

Rare L4 sublineages among drug-sensitive and multidrug-resistant strains, represented with less than 4 strains, were L4.3/T1-RUS2, L4.5/NEW1, L4.3/LAM5, L4/T1-T2, and L4.1.2/T4-CEU1. All L4.5/NEW1 isolates (*n* = 4) were MDR, which supports the previous observation that the NEW1 sublineage is prone to drug resistance [66].

## 4. Discussion

We have shown that currently 106 spoligotypes of Lineage 4 and two of L2/Beijingof M. tuberculosis circulate in Bulgaria. Historically, multiple introductions of different genotypes and lineages have occurred through immigration. How is the biodiversity of M. tuberculosis in Bulgaria related to immigration waves of Turkish and Gypsy ethnic groups, which are prevalent in the country? Which factors were dominant in the formation of biodiversity, the human genetic background, migration, or the ecological adaptation of imported genotypes?

**Figure 2:**
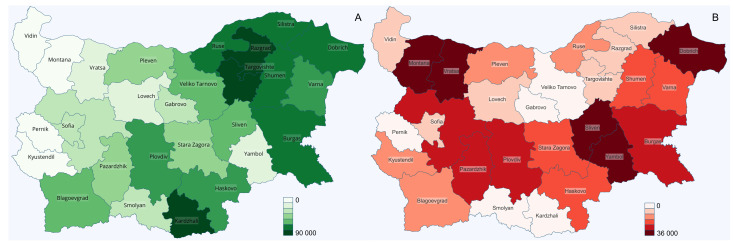
Ethnic maps of Bulgaria: (**A**) administrative districts and numbers of Turkish population; (**B**) administrative districts and numbers of Gypsy population.

The ethnic map of Bulgaria in 2011 showed that 1.1–1.5 million Turks and Gypsies live in the country. The two ethnic groups are not evenly distributed across the country (Figure 2). Persons who identify themselves as Turkish are located in several districts, including Kardzhali, Razgrad, Targovishte, Shumen, Silistra, Dobrich Ruse, and Burgas, where (in total) 63.7% of them live (Figure 2A). Persons from the Roma ethnic group are present in all districts. The biggest proportions of Gypsy ethnicity is in the Montana district (12.7%) and the Sliven district (11.8%), followed by the Dobrich district (8.8%) and the Yambol district (8.5%), while the proportion of Gypsies in the entire country is 4.9% (Figure 2B) [33].

**Figure 3:**
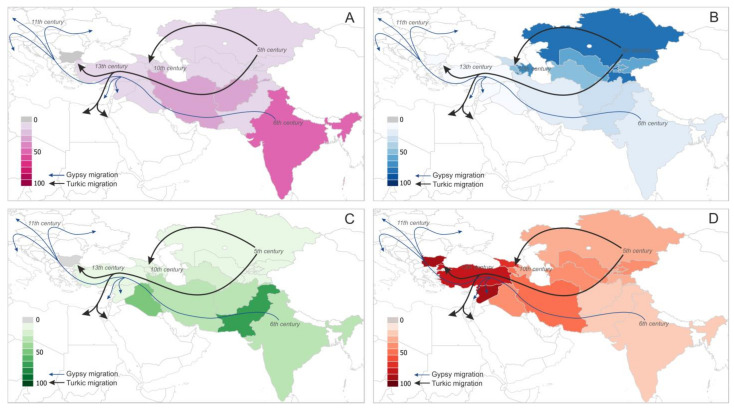
Migration roads of Gypsy communities from the Indian subcontinent and Turkic peoples from Central Asia to Europe, and the prevalence of *M. tuberculosis* lineages L1-L4 on the map of modern states: (**A**) prevalence of L1; (**B**) prevalence of L2; (**C**) prevalence of L3; and (**D**) prevalence of L4.

The MIRU-VNTR and spoligotyping methods have proven capacities to correctly identify and discriminate between MTBC lineages. Our ten-year studies confirm the absence of the *M. tuberculosis* genotypes L3/CAS and L1/EAI sublineages in Bulgaria, which are widespread in Northern India, Pakistan, Afghanistan, Iran, and Iraq [12,47,62,63,67,68,69]. Northwest India is the presumed geographical area of the Gypsy communities that migrated to Europe 10 centuries ago, crossing Pakistan, Afghanistan, Iran, Iraq, and Turkey (Figure 3). 

It is well documented that immigrants with active or latent tuberculosis bring with them MTBC genotypes from their countries of origin. Inevitably, Gypsies who immigrated to Bulgaria 10 centuries ago brought new lineages and genotypes from their geographic regions of origin in India. In Bulgaria, 4.9% to 9% of the total population are Gypsies and 8.8% are Turks [33]. It can be assumed that at least 14% to 16% of the MTBC genotypes should resemble those of Indian or Middle East origin. Where are they now?

Our study was limited in identifying the ethnic origin of patients. When hospitalized in Bulgaria, patients are not required to declare an ethnicity. Patients are registered only by name, date of birth, place of birth, personal identification number, and current home address. All Bulgarian citizens have both Bulgarian nationality and EU citizenship. Voluntary information on the ethnicity of citizens is collected only during the national census, which is conducted once every ten years. The last national census, conducted in 2011, showed that 96.6% (564,858) of the Turkish ethnic group identified Turkish as their mother tongue, while 3.2% (18,975) declare Bulgarian as their mother tongue. In the Gypsy ethnic group, the Roma language was identified as the mother tongue by 85% (272,710), while 7.5% (24,033) identified Bulgarian as their mother tongue, 6.7% (21,440) identified Turkish as their mother tongue, and 0.6% identified Romanian as their mother tongue [33].

It was not possible to determine ethnicity by name alone. Some Gypsies have Bulgarian names; some speak Turkish and have Turkish names. Persons of Turkish ethnicity live mostly in compact communities in the south of the country, in the Arda river basin, in the district of Kardzhali, or in the northeastern part of the country. Ethnic Turks have traditionally had Turkish names, but in 1984–1985 a name-changing campaign was begun by the Bulgarian government. As a result, ethnic Turks were registered with Bulgarian names. Meanwhile, about 350,000 ethnic Turks emigrated from Bulgaria to Turkey in 1989, during the so-called forced “big excursion.” The Turkish government then changed their names from Bulgarian to Turkish. 

With the democratic changes in the 1990s, the Bulgarian government decided to make it the possible for all ethnic Turks to return to their original names, and dual citizenship was allowed. Some Turks kept their Bulgarian names, while others decided to keep their Turkish names while in Turkey and their Bulgarian name in Bulgaria, with a Bulgarian ID card. 

Yet another minority group in Bulgaria, comprised of 130,000 people, are the Pomaks, who are Bulgarian Muslims with Bulgarian or Turkish-Arab names. 

Because of these numerous alternatives for names, it was not possible to identify an individual patient’s ethnicity in this study.

Elena Marushiakova and Vesselin Popov, from the Institute of Ethnology and Folklore Studies at the Ethnographic Museum at the Bulgarian Academy of Sciences, are currently carrying out major research work among the Gypsy communities in the Balkans and Central Europe. Their conclusions indicate that Gypsy groups lead a conservative, closed lifestyle, isolated from the rest of the population [27,28]. Importantly, Gypsies are the poorest minority group in Bulgaria and within the EU. According to the Bulgarian National Tuberculosis Control Programme, the state of health of Gypsy communities is characterized by higher morbidity, lower life expectancy, and higher mortality. Gypsies are a high priority group, but one of the most difficult groups to reach. In 2013, tuberculosis among Gypsies ranged from 60 cases (in all forms of tuberculosis) per 100,000 people in the Plovdiv district to 476 per 100,000 in the Vratsa district, which represents a range of 2.4% to 19% of reported TB cases among the general population. Nearly half of the patients with MDR are from the Gypsy community. A similar situation exists in North Macedonia and Romania. The incidence of tuberculosis among the Gypsy community is 3 to 15 times higher than for the general population [70,71]. 

Among the Gypsies, there is a law of ritual purity, which means “not to touch others” [49]. Gypsies exist as highly closed community groups, and over time the genotypes of *M. tuberculosis* that Gypsies may have brought with them during their settlement in Bulgaria may have disappeared (Figure 3). 

The local ecological landscape or the local presence of other MTBC isolates may have been major drivers of biodiversity in the process of transmitting existing or of newly imported genotypes. If the L1 and L3 genotypes were present in the ancestors of today’s Bulgarian Gypsies, we may conclude that the imported genotypes of *M. tuberculosis* (L3/CAS and L1/EAI) may have been counter-selected because they possessed low adaptive capacity in the local ecology and environment or, more likely, founder effects suggest that these historical populations were not infected by L1 or L3 *M. tuberculosis* sublineages. If so, it would be clear that Gypsy immigrants did not spread any specific genotypes of *M. tuberculosis* EAI and CAS in Bulgaria, which are common in today’s northwestern India and Pakistan. The recent demographical division in Pakistan and Indian demography may explain such a paradoxical situation. However, there is also some evidence that the L1/EAI and L3/CAS sublineages were already in circulation by the time Gypsies started their migrations [72].

The prevalence of the L2/Beijing sublineage in Bulgaria is low, while in the Inner Asia territories of Mongolia, Kazakhstan, Turkmenistan, Azerbaidzhan, Armenia, and Giorgia its prevalence is significant [1,12,47,63,66,67,69,72,73]. Turkic tribes started migrations from Inner Asia, crossing the Caspian Sea region, and in the 13th century they established the Ottoman State in the territory of modern Turkey. During the next several centuries, they planned active military campaigns in Europe (Figure 3). Currently in Bulgaria and Turkey, common genotypes of *M. tuberculosis* can be identified with geographical specificity. The Turkish and Bulgarian genetic MTBC landscape remains dominated by L4, and more specifically by L4.2. Less than 1% of the genotypes of the L1/EAI, L2/Beijing, and L3/CAS sublineages are found in Turkey [12,74,75,76,77]. At the same time, Bulgarian Turks are not carriers of the CAS and EAI genotypes, although each year several hundred thousand Turks visit each other from both sides of the border. 

After the collapse of the Ottoman Empire, Bulgarian Turks remained living in the territory of Bulgaria, and they have been present in the Balkan Peninsula for seven centuries. After so many centuries of presence, it is possible that some of the genotypes of M. tuberculosis imported by the Turks in Bulgaria did not adapt and were replaced. This may have included all potential strains of the CAS and EAI sublineages that had adapted ecologically and diffused in Turkey, but not in Bulgaria. 

The low incidence of the CAS, EAI, and Beijing sublineages in Turkey leads to the logical supposition that lineages L1–L3 were somehow ecologically limited and did not spread in this region of the Middle East [68,74,75,76,77]. These discrepancies cannot be fully explained with a specific human genetic background. In Azerbaijan, the L2/Beijing sublineage is dominant, but it is almost absent in Turkey. Azeri people share a very close historical past, language, and human genetic background with modern Turks. Trade, political, cultural, border, and tourist ties between the two countries are very active. Moreover, the territory of modern Turkey has been a main migration road between Asia and Europe since ancient times. A potential explanation for why the L2/Beijing sublineage remains rare in Turkey is that some environmental factors may play a role in MTBC adaptation.

There have been several documented migration waves of Turks to Bulgaria [52,53,54,55,56]. The Turkish historian Ilber Ortayli reports that Sultans Fatih and Mahmud II settled in Southern Bulgaria in 1440 and 1820 with Turks from Central Turkey. In 1514, Sultan Selim I defeated the Iranian Shah Ismail. The Turks, who supported Shah Ismail were deported to Northern Bulgaria. During the expansion of the Ottoman Empire to the West in the 15th century to the 17th century, the city of Edirne was a center where troops gathered. Historically, military campaigns for the conquest of Europe started in the early spring. For two centuries, huge military units marched back and forth through Sofia, Belgrade, Budapest, andVienna in the spring and late autumn. The main participants were Ottomans from Asia Minor, but there were also military units from North Africa. At least 100,000 soldiers and civilians took part in military campaigns each year. In the 17th and 18th centuries, a significant number of Arabs participated in the campaigns against the Kingdom of Poland, in the southern Russian steppes, and in the Crimean Peninsula. Their passage was through Bulgaria. During the Russo-Turkish War in 1877–1878, also known as the War of Liberation for Bulgaria, Moors (Mauri) from Africa were also involved on the Balkan front. Historical sources indicate that in around 1520, the Turks constituted about 20% of the population of Bulgaria. In 1875, they represented 50%. Soon after the Russian-Turkish War in 1881, they declined to 20–23%, following mass migration. Bulgarians living in Turkey (>100,000 people, only in Istanbul) migrated back to Bulgaria.

In 1915 and 1916, about 22,000 Armenians from Turkey moved to Bulgaria [57]. From 1917 to 1922, there was civil war in Russia. Between 1920 and 1922, 32,000 to 34,000 Russian soldiers and their families temporarily settled in Bulgaria [58]. Since 1975, there have been about 7000 African, 17,000 Arab, and >15,000 Vietnamese students and workers in the country. The census conducted in Bulgaria in 2011 showed that 36,700 foreigners live in the country, and that every second foreigner was a citizen of the EU Sixty five percent of the non-EU citizens were Russians, 16.5% were Ukrainian, and 5% were from Moldova. The L2/Beijing sublineage is prevalent in Russia, the Ukraine, Moldova, Armenia, and Vietnam.

In a nationwide study of 197 drug-susceptible isolates obtained during 2003–2004, no isolates of the Beijing sublineage were identified in Bulgaria [78]. A subsequent study during 2004–2006 also failed to identify the Beijing genotype among 133 drug-resistant and drug-susceptible *M. tuberculosis* isolates. [79]. As previously mentioned, the first L2/Beijing strains introduced in Bulgaria were during 2007–2009. The role of migraions in the L2 diffusion in Bulgaria was confirmed.

After the political changes that started in 1989, a significant number of Bulgarians emigrated around the world. According to Bulgarian diplomatic missions, in 2011 no less than 2,018,000 Bulgarians were living in 70 countries. In 2020, Bulgarians who travelled abroad numbered 3,973,009, Many of whom live abroad permanently. Inevitably, a small part of this huge mass of people was exposed to and affected by tuberculosis.

The geographical restriction of lineages as a function of coevolution of the pathogen within local human populations is an oft-stated observation [47,80,81,82]. However, the consequence of human migrations should be weighed against local demographical histories. In our study, we tried to provide a broader understanding of the complexity of such phenomena. We reporedt historical evidence that, in the long term, newly imported genotypes could have been constrained to adapt to the local environment, faced with other bacilli that were already present and more fit. We hypothesized that if imported MTBC genotypes did not adapt, they may gradually go out of circulation and be lost. For a permanent and sustained tranmission, imported MTBC genotypes could require a long period for ecological adaptation to local ecosystems and favorable demographic conditions. 

A new incoming ethnical group with its own genetic background may need to adapt to new MTBC genotypes, which are different from those in their place of origin. In support of this hypothesis are the empirical data reported in our study. Strains of the L1/EAI and L3/CAS sublineages may not adapt to the local ecological and environmental factors found in Bulgaria. Lineage 4 is geographically widespread and considered more virulent than other lineages that are more geographically restricted. Increased virulence is associated with delayed or reduced pro-inflammatory host immune responses, greater severity of disease, and enhanced transmission [80]. At the same time, some L4 sublineages may only be geographically restricted, i.e., the L4/TUR sublineage is found in Albania, Serbia, Bulgaria, and Turkey [60,61,75]. The introduction and spread of L4/TUR in the Balkan Peninsula could have happened during the invasion and presence of the Ottoman turks.

The ecological adaptation of imported *M. tuberculosis* genotypes remains an open fundamental question. A recent study demonstrated that, on a global scale, L1 and L3 are less transmissible, compared to L2 and L4 [46]. We extend this conclusion by adding that L1 and L3 might be less ecologically adaptive to new geographic environments. On a global scale, it could be observed that geographical restriction and a sharp decline of L1 and L3 *M. tuberculosis* genotypes, from east to west, could be correlated with the environmental factor rather than with the genetic background of Gypsies or other migration groups in their migrations to the west (Figure 3 and Table S1).

Our results suggest that the genetic background of the local population in Bulgaria was not the main driver for biodiversity and adaptation of introduced *M. tuberculosis* genotypes. Bulgarian tribes settled in the territory of the Balkan Peninsula in the 7th century AD. Before that, they lived and migrated in territories from in Inner Asia to the southern Russian steppes, where it is likely that other *M. tuberculosis* lineages and sublineages circulated. It is unlikely that all the genotypes they brought to this new geographic area, the Balkan Peninsula, have ecologically adapted. Our empirical data and the historical facts attempt to provide more evidence that could substantiate these claims. Undoubtedly, future research efforts will need to be directed to the development of locally specific schemes for the treatment of tuberculosis, focusing on the genotype and the development of more effective topical vaccines.

## 5. Conclusions

Migration and the permanent establishment of large masses of people in new geographical areas are considered as the major factors in the global spread of infections around the world. These events give rise to emerging infections in given geographical areas.

The waves of human immigration to Bulgaria in the last 10 centuries could have played a limited role in the formation of the genetic biodiversity of MTBC in the country, if ecological adaptation was the norm for bacterial pathogen replacement. The main factors for genetic biodiversity in Bulgaria could have been the relative fitness and the adaptability of imported genotypes of MTBC to ecological and other unknown environmental parameters. Competitive displacement or competitive exclusion of imported genotypes may have played an essential role in shaping today’s observed MTBC structure. In this sense, the hypothesis of specific local environmental factors for the adaptation and biodiversity of the genotypes of MTBC in Bulgaria has a sound basis. 

In the long run, imported MTBC genotypes would be lost if they did not adapt to local environmental factors. This could be a possible scenario to explain the absence of MTBC lineages in Bulgaria that were likely to have been common in the places of origin of the migrating Turkic and Roma ethnic groups, even though founder effects are also likely. Our empirical data and analysis of migrations could give support to the hypothesis that if the human genetic background has a demonstrated role in host-pathogen interactions, it could also be complemented by other unknown environmental factors affecting the fitness of new genotypes in new environments. The ecological adaptability of MTBC genotypes could depend on multifactorial environmental factors that have not been studied, and that would complement the already complex host-pathogen matrix.

The sustainable ecological adaptation of MTBC genotypes may rely on still-undescribed mechanisms or factors that afffeted the efficiency of tuberculosis transmission during previous migrations.

## Figures and Tables

**Table 1:** Identified spoligotype lineage and sublineages as defined in the SITVIT2 database and inferred lineage and sublineage WGS-naming. When identical spoligotype signatures cover different sublineages, they are reported when known. Number of identified strains and prevalence in percentage among drug-sensitive (DS) and multidrug-resistant (MDR) MTBC sublineages in Bulgaria for the period 2008–2018.

Sublineage	DS, *n* (%)	DS, 95% CI	MDR, *n* (%)	MDR, 95% CI
L2/Beijing	5 (0.76)	0.1–1.43	13 (3.37)	1.57–5.17
L4/L4.1/T1	274 (41.83)	38.05–45.61	105 (27.2)	22.76–31.64
L4.2.2.1/TUR	5 (0.76)	0.1–1.43	199 (51.55)	46.56–56.54
L4.4/S	144 (21.98)	18.81–25.16	25 (6.48)	4.02–8.94
L4.1.2/H1	43 (6.56)	4.67–8.46	9 (2.33)	0.82–3.84
L4.1.2/L4.5/H3	41 (6.26)	4.4–8.11	6 (1.55)	0.32–2.78
L4.4/L4.3/S	33 (5.04)	3.36–6.71	6 (1.55)	0.32–2.78
L4.1.2/X1	23 (3.51)	2.1–4.92	2 (0.52)	−0.2–1.24
L4.10/T3	17 (2.6)	1.38–3.81	1 (0.26)	−0.25–0.77
L4/H3–T3	8 (1.22)	0.38–2.06	10 (2.59)	1.01–4.17
L4.6/L4.10/T2–T3	8 (1.22)	0.38–2.06	4 (1.04)	0.03–2.05
L4.2/URAL	11 (1.68)	0.7–2.66	0	-
L4.3.4.2/LAM11–ZWE	9 (1.37)	0.48–2.27	0	-
L4.3/LAM9	7 (1.07)	0.28–1.86	0	-
L4.6/T2	6 (0.92)	0.19–1.65	0	-
L4.3/LAM3	5 (0.76)	0.1–1.43	0	-
L4.1.2.1/H2	4 (0.61)	0.01–1.21	0	-
L4.3/T1–RUS2	3 (0.46)	−0.06–0.98	1 (0.26)	−0.25–0.77
L4.5/NEW1	0	-	4 (1.04)	0.03–2.05
L4.1.2/T4–CEU1	2 (0.31)	−0.85	1 (0.26)	−0.25–0.77
L4.3/LAM5	2 (0.31)	−0.12–0.73	0	-
L4/T1–T2	2 (0.31)	−0.12–0.73	0	-
L6/BOV1	3 (0.46)	−0.06–0.98	0	-
Total (*n* = 1041)	655	-	386	-
χ^2^(df), P	2763.73 *** (21)	-	1489.56 *** (13)	-

Significance level of Pearson’s χ^2^ test, *p* < 0.001 (***).

**Table 2:** Probability for multidrug resistance of dominant sublineages in Bulgaria for the period 2008–2018. OR ≥ 1 is in bold.

Sublineage	MDR, *n*	Total, *n*	%	95% CI	OR	χ^2^(df = 1)
L2.2/Beijing	13	18	72	51.31–92.69	**2.60**	3.56 ^T^
L4.1/T1	105	379	28	23.49–32.51	0.38	75.36 ***
L4.1/T1-SIT 53	49	129	38	29.62–46.38	0.61	7.45 **
L4.1/T1-SIT 154	8	44	18	6.6–29.4	0.22	17.82 ***
L4.1/T1-SIT 284	28	56	50	36.9–63.1	**1.00**	0.00
L4.2.2.1/TUR	199	204	98	95.88–100	**39.80**	184.49 ***
L4.4/S	25	169	15	9.65–20.35	0.17	83.79 ***
L4.4/S-SIT 34	4	51	8	0.62–15.38	0.09	36.26 ***
L4.4/S-SIT 125	21	110	19	11.66–26.34	0.24	42.04 ***

Significance level of Pearson’s χ^2^ test, *p* < 0.001 (***), *p* < 0.01 (**), 0.05 < *p* < 0.06; (T)—trends towards significance.

## Data Availability

Data are available as Supplementary Materials. Datasets of drug-sensitive and multidrug-resistant *M. tuberculsis* strains from Bulgaria were deposited in a publicly available database: zenodo.org, https://zenodo.org/record/5801311, accessed on 15 November 2021.

## Supplementary Materials

The following supporting information can be downloaded at: https://www.mdpi.com/article/10.3390/microorganisms10010146/s1, Figure S1: Number of TB cases and notification rates in Bulgaria, 2008–2018; Table S1: Dominant *M. tuberculosis* lineages throughout the geographic regions (modern countries) crossed by the Gypsies and the Turks during their migration to Bulgaria and Europe.
