# Supplementary material for: Biodiversity of Mycobacterium tuberculosis in Bulgaria Related to Human Migrations or Ecological Adaptation
Source: Microorganisms. 2022 Jan 11;10(1):146. doi: 10.3390/microorganisms10010146 (PMC8778017; doi:10.3390/microorganisms10010146)
Supplement: Supplementary file 1 [file microorganisms-10-00146-s001.zip › microorganisms-1547137-supplementary.pdf]

Table S1. Dominant *M. tuberculosis* lineages throughout the geographic regions (modern countries) crossed by the Gypsies and the Turks during their migration to Bulgaria and Europe.

Gypsy migration	Dominant Lineages	Ref.	Migration of the Turks	Dominant Lineages	Ref.
India	L1/EAI-45%<br>L2/Beijing-7%<br>L3/CAS-22%<br>L4 - 8%	1, 2, 6, 9, 14	Kazakhstan	L2/Beijing-80%<br>L4-20%	9, 13, 15
Pakistan	L1/EAI-10%<br>L3/CAS-65%<br>L4-8%	1, 2, 6, 9	Turkmenistan	L2/Beijing-50%<br>L3/CAS-7%<br>L4-40%	1, 9, 15
Afghanistan	L3/CAS-28%	2, 3, 6, 9	Kyrgyzstan	L2/Beijing-60%<br>L4-40%	16,18
Iran	L1/EAI-13%<br>L2/Beijing-8%<br>L3/CAS-19%<br>L4/45%	1, 2, 3, 4, 6, 9	Azerbaijan	L2>50-70%<br>L4-20%	1, 9
Iraq	L3/CAS-41%<br>L4-39%	1, 2, 5, 9	Georgia	L1/EAI-2%<br>L2/Beijing-26%<br>L4-70%	1, 8, 9
Syria	L4>90%	1, 17	Iran	L1/EAI-13%<br>L2/Beijing-8%<br>L3/CAS-19%<br>L4/45%	1, 2, 3, 6, 9
Turkey	L4>95%<br>L1/EAI-1%; L2/Bei-jing-1%; L3/CAS – 1%	1,3,7,9,10, 11,12,19	Iraq	L3/CAS-41%<br>L4-39%	1, 2, 5, 9
Bulgaria	L4-99%<br>L2/Beijing-1%	This study	Syria	L4>90%	1, 17

References:

1. Couvin, D.; David, A.; Zozio, T.; Rastogi, N. Macro-geographical specificities of the prevailing tuberculosis epidemic as seen through SITVIT2, an updated version of the *Mycobacterium tuberculosis* genotyping database. *Infect Genet Evol* **2018,** *72*, 31-43. doi: 10.1016/j.meegid.2018.12.030.
2. Couvin D, Reynaud Y, Rastogi N. Two tales: Worldwide distribution of Central Asian (CAS) versus ancestral East-African Indian (EAI) lineages of Mycobacterium tuberculosis underlines a remarkable cleavage for phylogeographical, epidemiological and demographical characteristics. PLoS One. 2019 Jul 12;14(7):e0219706. doi:
3. Merza, M. A.; Farnia, P.; Salih, A. M.; Masjedi, M. R.; Velayati, A. A. The most predominant spoligopatterns of Mycobacterium tuberculosis isolates among Iranian, Afghan-immigrant, Pakistani and Turkish tuberculosis patients: a comparative analysis. Chemotherapy. **2010**, 56(3), 248-257. doi: 10.1159/000316846.
4. Hadifar, S.; Fateh, A.; Pourbarkhordar, V.; Siadat, S. D.; Mostafaei, S.; Vaziri, F. Variation in M. tuberculosis population structure in Iran: a systemic review and meta-analysis. BMC Infect Dis. **2021**, 4, 21. doi: 10.1186/s12879-020-05639-7
5. Ahmed, M. M.; Mohammed, S. H.; Nasurallah, H. A.; Ali, M. M.; Couvin, D.; Rastogi, N. Snapshot of the genetic diversity of Mycobacterium tuberculosis isolates in Iraq. *Int J Mycobacteriol* **2014**, 3(3), 184-196. doi: 10.1016/j.ijmyco.2014.07.006.
6. Netikul, T.; Palittapongarnpim, P.; Thawornwattana, Y.; Plitphonganphim, S. Estimation of the global burden of Mycobacte-rium tuberculosis lineage 1. *Infect Gen Evol* **2021**, 91, 104802. https://doi.org/10.1016/j.meegid.2021.104802.
7. Karagoz, A.; Tutun, H.; Altintas, L.; Alanbayi, U.; Yildirim, D.; Kocak, N. Molecular typing of drug-resistant Mycobacterium tuberculosis strains from Turkey. *J Glob Antimicrob Resist* **2020**, 23, 130-134, https://doi.org/10.1016/j.jgar.2020.08.012.
8. Niemann, S.; Diel, R.; Khechinashvili, G.; Gegia, M.; Mdivani, N.; Tang, Y. W. Mycobacterium tuberculosis Beijing lineage favors the spread of multidrug-resistant tuberculosis in the Republic of Georgia. *J Clin Microbiol* **2010**, 48, 3544-3550. doi: 10.1128/JCM.00715-10.

9. O'Neill, M.; Shockey, A.; Zarley, A.; Aylward, W.; Eldholm, V.; Kitchen, A.; Pepperell, C. Lineage specific histories of Mycobacterium tuberculosis dispersal in Africa and Eurasia. *Mol Ecol* **2019**, 28(13), 3241-3256. doi: 10.1111/mec.15120.
10. Gencer, B.; Shinnick, T. M. Molecular genotyping of Mycobacterium tuberculosis isolates from Turkey. *Am J Infect Dis* **2005**, 1, 5-11.
11. Durmaz, R.; Zozio, T.; Gunal, S.; Yaman, C.; Cavusoglu, A.; Guney, C.; et al. Genetic diversity and major spoligotype families of drug-resistant Mycobacterium tuberculosis clinical isolates from different regions of Turkey. *Infect Genet Evol* **2007**, 7, 513-519, https://doi.org/10.1016/j.meegid.2007.03.003.
12. Kisa, O.; Tarhan, G.; Gunal, S.; Albay, A. Durmaz, R.; Saribas, Z.; et al. Distribution of spoligotyping defined genotypic lineages among drug-resistant Mycobacterium tuberculosis complex clinical isolates in Ankara, Turkey. *PLoS ONE* **2012**, 7(1): e30331. https://doi.org/10.1371/journal.pone.0030331.
13. Klotoe, B.J.; Kacimi, S.; Costa-Conceicão, E. l.; Gomes, H.M.; Barcellos, R.B.; Panaiotov, S.; Haj Slimene, D.; Sikhayeva, D.; Sengstake, S.; Schuitema, A.R.; Akhalaia, M.; Alenova, A.; Zholdybayeva, E.; Tarlykov, P.; Anthony, R.; Refregier, G.; Sola, C. Genomic characterization of MDR/XDR-TB in Kazakhstan by a combination of high-throughput methods predominantly shows the ongoing transmission of L2/Beijing 94–32 central Asian/Russian clusters. *BMC Infect Dis* **2019**, 19, 553. https://doi.org/10.1186/s12879-019-4201-2.
14. Poonawala, H.; Kumar, N.; Peacock, S.J. A review of published spoligotype data indicates the diversity of Mycobacterium tuberculosis from India is under-represented in global databases. *Infect Genet Evol* **2020**, 78:104072. doi: 10.1016/j.meegid.2019.104072.
15. Cox, H. S.; Kubica, T.; Doshetov, D.; Kebede, Y.; Gerdess, S.R.; Niemann, S. The Beijing genotype and drug resistant tuberculosis in the Aral Sea region of Central Asia. *Respir Res* ***2005,*** 6, 134, https://doi.org/10.1186/1465-9921-6-134.
16. Mokrousov, I.; Isakova, J.; Valcheva, V.; Aldashev, A.; Rastogi, N. Molecular snapshot of Mycobacterium tuberculosis population structure and drug-resistance in Kyrgyzstan. *Tuberculosis (Edinb)* **2013,** 93(5), 501-507. doi: 10.1016/j.tube.2013.05.008.
17. Zarziur, H.; Madania, A.; Ghoury, I.; Habous, M. High resolution genotyping of M. tuberculosis isolates from Syria using MIRU-VNTR. *Biomed Biotechnol Res J,* **2019**, *3, 1-8,* DOI: 10.4103/bbrj.bbrj_139_18.
18. Engström, A.; Antonenka, U.; Kadyrov, A.; Kalmambetova, G.; Kranzer, K.; Merker, M.; Kabirov, O.; Parpieva, N.; Rajabov, A.; Sahalchyk, E.; Sayfutdinov, Z.; Niemann, S.; Hoffmann, H. Population structure of drug-resistant *Mycobacterium tuberculosis* in Central Asia. *BMC Infect Dis* **2019**, 19, 908, https://doi.org/10.1186/s12879-019-4480-7
19. Zozio, T.; Allix, C.; Gunal, S.; Saribas, Z.; Alp, A.; Durmaz, R.; Fauville-Dufaux, M.; Rastogi, N.; Sola, C. Genotyping of Mycobacterium tuberculosis clinical isolates in two cities of Turkey: Description of a new family of genotypes that is phylogeographically specific for Asia Minor. *BMC Microbiol* **2005**, 5, 44, https://doi.org/10.1186/1471-2180-5-44.

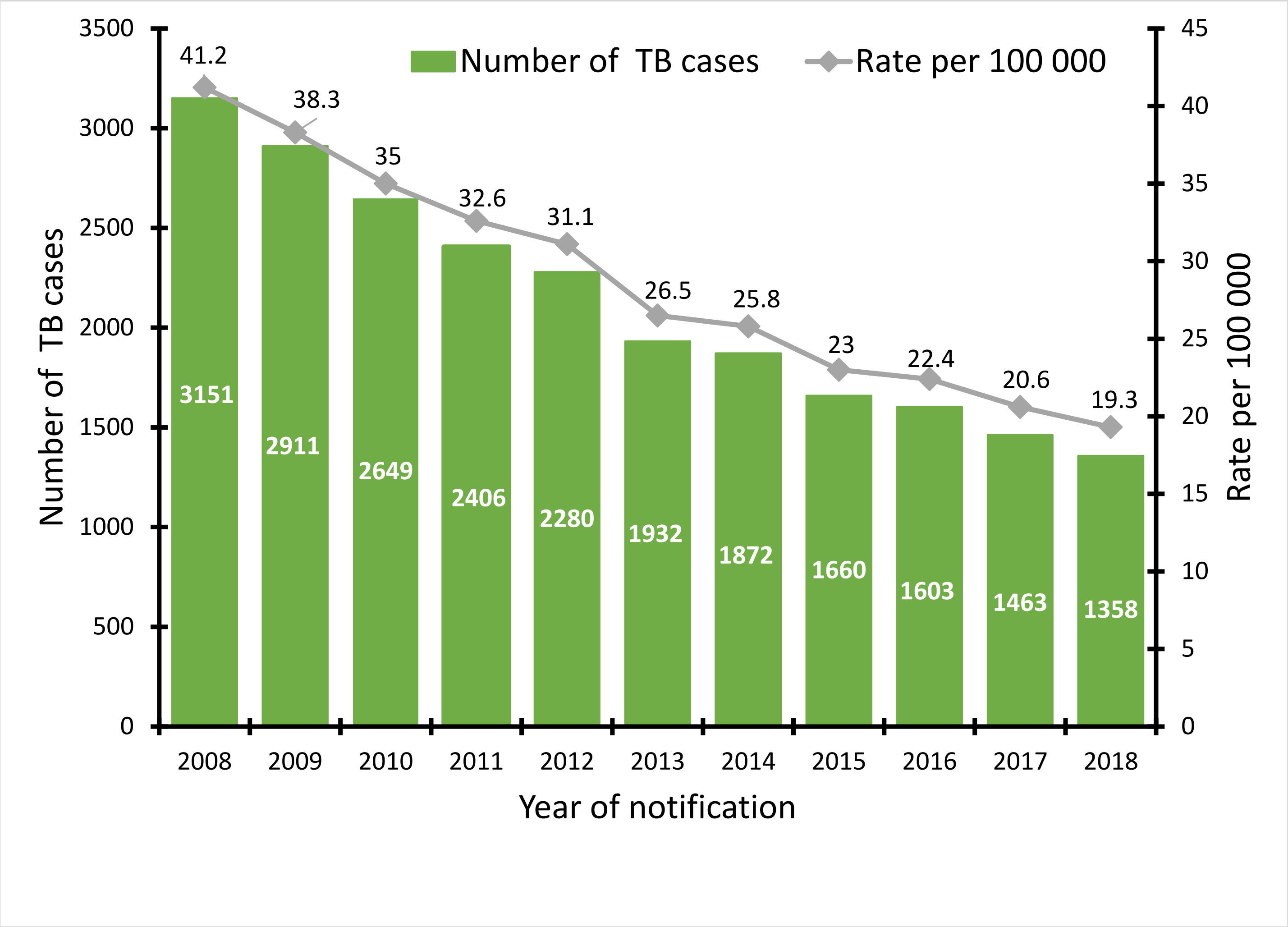

Figure S1. Number of TB cases and notification rates in Bulgaria, 2008–2018.
